# Analysis of long-term oncological results of clinical versus pathological responses after neoadjuvant treatment in locally advanced rectal cancer

**DOI:** 10.1186/s12957-020-02094-1

**Published:** 2020-11-30

**Authors:** Mariana F. Coraglio, Martin A. Eleta, Mirta R. Kujaruk, Javier H. Oviedo, Enrique L. Roca, Guillermo A. Masciangioli, Guillermo Mendez, Ilma S. Iseas

**Affiliations:** 1 Colorectal Surgery Unit, Gastroenterology Hospital, Dr. Carlos Bonorino Udaondo, Av. Caseros 2061, 1264 Ciudad Autónoma de Buenos Aires (CABA), Argentina; 2 Imaxe Image Diagnosis Center, Ciudad Autónoma de Buenos Aires (CABA), Argentina; 3 Pathology Unit, Gastroenterology Hospital, Dr. Carlos Bonorino Udaondo, Ciudad Autónoma de Buenos Aires (CABA), Argentina; 4 Coloproctology Fellowship National Health Cancer Institute, Gastroenterology Hospital, Dr. Carlos Bonorino Udaondo, Ciudad Autónoma de Buenos Aires (CABA), Argentina; 5 Oncology Unit, Gastroenterology Hospital, Dr. Carlos Bonorino Udaondo, Ciudad Autónoma de Buenos Aires, Argentina

**Keywords:** Rectal cancer, Clinical complete response, Pathological complete response, Neoadjuvant treatment, Watch and wait

## Abstract

**Background:**

Nonoperative management after neoadjuvant treatment in low rectal cancer enables organ preservation and avoids surgical morbidity. Our aim is to compare oncological outcomes in patients with clinical complete response in watch and wait strategy with those who received neoadjuvant therapy followed by surgery with a pathological complete response.

**Methods:**

Patients with non-metastatic rectal cancer after neoadjuvant treatment with clinical complete response in watch and wait approach (group 1, *n* = 26) and complete pathological responders (ypT0N0) after chemoradiotherapy and surgery (group 2, *n* = 22), between January 2011 and October 2018, were included retrospectively, and all of them evaluated and followed in a multidisciplinary team. A comparative analysis of local and distant recurrence rates and disease-free and overall survival between both groups was carried out. Statistical analysis was performed using log-rank test, Cox proportional hazards regression model, and Kaplan-Meier curves.

**Results:**

No differences were found between patient’s demographic characteristics in both groups. Group 1: distance from the anal verge mean 5 cm (*r* = 1–12), 10 (38%) stage III, and 7 (27%) circumferential resection margin involved. The median follow-up of 47 months (*r* = 6, a 108). Group 2: distance from the anal verge mean 7 cm (*r* = 2–12), 16 (72%) stage III, and 13 (59%) circumferential resection margin involved. The median follow-up 49.5 months (*r* = 3, a 112). Local recurrence: 2 patients in group 1 (8.3%) and 1 in group 2 (4.8%) (*p* = 0.6235). Distant recurrence: 1 patient in group 1 (3.8%) and 3 in group 2 (19.2%) (*p* = 0.2237). Disease-free survival: 87.9% in group 1, 80% in group 2 (*p* = 0.7546). Overall survival: 86% in group 1 and 85% in group 2 (*p* = 0.5367).

**Conclusion:**

Oncological results in operated patients with pathological complete response were similar to those in patients under a watch and wait strategy mediating a systematic and personalized evaluation. Surgery can safely be deferred in clinical complete responders.

## Introduction

Total mesorectal excision (TME) surgery has been the historical standard of care for the cure of rectal cancer. Neoadjuvant chemoradiotherapy (CRTn) was incorporated a few decades ago as an additional and valuable tool to treat locally advanced tumors. This therapy can achive a better local control of the disease [1, 2] and clinical or pathological complete response. Habr-Gama et al. [3] and other groups of researchers described acceptable long-term oncological results with a non-operative management called watch and wait (WW) in patients with clinical complete response (cCR), similar to those observed in patients with pathological complete response (pCR) after surgical resection. Several international series reflect variable rates of complete response, ranging from 2 to 78.4% of cCr and from 6.5 to 30% of pCR [4–12]. Furthermore, the complete response after CRTn represents a surrogate endpoint for improved overall survival [4, 10, 13].

One of the controversial issues of this pathological entity is sphincter preservation, which is still impossible in up to 50% of low rectum tumor cases, causing a negative impact on the physical and emotional sphere as well as on the quality of life of patients and even their families [1, 3, 6].

The WW strategy is increasingly accepted as it allows organ preservation and avoids surgical morbidity and mortality; however, despite the large number of publications, it is under constant evaluation and continues to be considered a controversial topic [9, 14–17].

The aim of this study is to evaluate whether the oncological results after CRTn followed by surgery in patients with rectal cancer and pCR are better than those in patients with clinical complete response under a non-operative strategy.

## Materials and methods

**Fig. 1:**
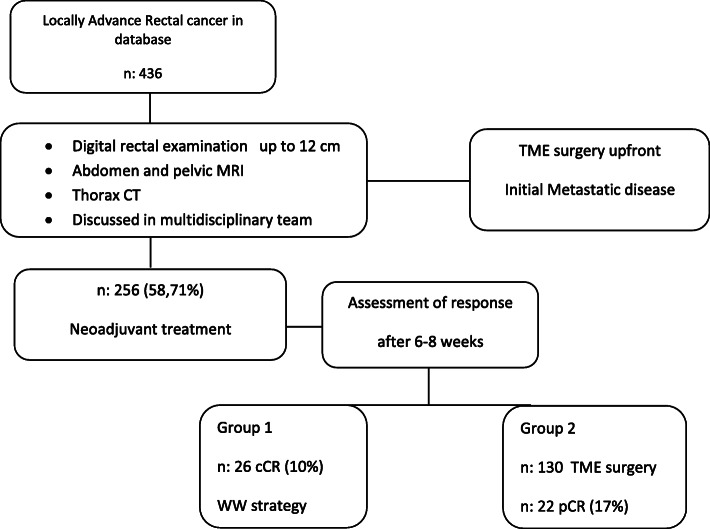
Selection of patients

Between January 2011 and October 2018, 256 patients with rectal adenocarcinoma who underwent CRTn were consecutively registered in a prospective database (58.71% of the patients with rectal cancer who were evaluated for treatment), 26 of whom achieved cCR and remained under the WW strategy and 130 underwent TME, 22 of whom achieved pCR (Fig. 1).

Inclusion criteria for our study were as follows:

1. Diagnosis of adenocarcinoma of the rectum located up to 12 cm from the anal verge in proctological exam presenting clinical complete response after CRTn who remains in the WW approach or pathological complete response after CRTn and surgery.
2. Initial clinical staging with magnetic resonance imaging (MRI) complying with high resolution parameters (3-mm slice thickness, 16–20 cm FOV, a matrix of 256 × 256 minimum [18])
3. Chest computed tomography scan (CT) without contrast
4. Analyzed in “Co-recto,” that is our multidisciplinary team.

Patients with a distant metastatic disease (stage IV) at initial staging or at the end of the treatment were excluded.

The absence of clinically detectable residual tumor by proctologic and endoscopic examination was defined cCR, a flat white scar, small telangiectasias, no ulcer, no nodularity, and on imaging with MRI ymrT0 ymrN0. The absence of viable tumor cells after the complete histopathological examination of the resected surgical specimen was informed pCR (ypT0 ypN0). The WW approach is a non-operative modality for clinical complete responders to nQRT, with a systematic follow-up.

We decided WW strategy in patients with clinical complete response when a sphincter preservation surgery is not possible or the patient does not want to be operated under any circumstances.

Two groups were formed: group 1 (G1) included the 26 patients who remained under the WW strategy and group 2 (G2) included the 22 patients who underwent TME surgery, whose pathological report was pCR (ypT0N0).

At baseline, each patient must undergo a digital rectal examination and rigid proctosigmoidoscopy, abdominal MRI, high-resolution pelvic T2W MRI (including diffusion-weighted), and thorax TC scan. Selected cases required a positron emission tomography (FDG PET/CT) procedure. The colon was examined by videocolonoscopy (VCC), and in those cases where it was incomplete, a double contrast barium enema was performed.

The therapeutic management was decided at the weekly meetings of the multidisciplinary team (“Co-recto”). The implemented neoadjuvant treatment was chemotherapy with capecitabine at a daily dose of 825 mg/m^2^ every 12 h continuously every weekday and concurrent 3D pelvic radiation therapy ranging between 4500 and 5040 cGy (depending on the treating center). Induction chemotherapy was indicated with three cycles of XELOX in patients with initial risk factors in the MRI, such as extramural vascular invasion (EMVI+), suspicious lateral lymph nodes, or highly symptomatic. Adjuvant chemotherapy was implemented in patients who presented mesorectal and/or lateral lymph node disease at initial staging.

Assessment of tumor response was performed 6 or 8 weeks after completion CRTn with the same studies done at the beginning .

A minimum waiting time of 8 weeks post CRTn was established for those patients who underwent surgery either because they presented a residual lesion or could undergo sphincter preservation surgery. TME technique was performed. In very low rectal tumors, the procedure of choice was intersphincteric resection with transanal handswen anastomoses, whereas an abdominoperineal amputation was the option in other cases. The anastomotic sites within 6 cm or less from the anal verge were protected with a loop ileostomy.

The anatomopathological study of the surgical resection specimens was performed by following a standardized protocol of the Hospital Pathology Service. Macroscopically, the ulcer and/or fibrosis area was sectioned at 5 mm intervals and total embedded. To determine the presence of residual tumor, each block was examined with a single section level stained with hematoxylin-eosin. The degree of response to the preoperative therapy was assigned in accordance to the recommendations of the College of American Pathologists (CAP) [19]. The cases presenting absence of tumor cells were registered as ypT0 and ypN0 (Grade 0 Complete response). For the classification of the pathological stage, the 8th edition UICC TNM Edition Staging System was used [20, 21].

Follow-up of the patients under WW strategy included proctologic examination, abdomen and pelvic MRI, and carcinoembryonic antigen (CEA) at 3-month intervals during the first year, annual VCC, and thorax-abdomen-pelvis TC scan every 6 months; MRI for the second year every 4 months; and every 6 months for the 3rd year, continuing annually. After surgery, all patients were monitored at 3-month intervals with CEA, at least two complete CT; the first 3 years and VCC the first year, continuing according to the findings [3, 22–24].

### Statistical analysis

The recurrence rate or local regrowth, distant progression, and disease-free and overall survival were evaluated in each group to determine if there were long-term differences between the two strategies analyzed.

Follow-up time was determined from the moment when WW was decided in G1 patients and from the date of surgery in G2 group, in both groups after treatment was concluded and the complete response was confirmed.

The study design was retrospective and descriptive. Statistical significance was assessed using the log-rank test for local and distant recurrence and the Cox proportional hazards regression model for overall survival. Kaplan-Meir curves were used to evaluate survival. A *p* value less than 0.05 was considered statistically significant. The statistical analysis was performed using Medcalc 11.2.1.0 software.

## Results

Twenty six patients in non-operative management (G1) and 22 patients who underwent TME and pCR (G2) were included, a total of 48 patients with complete response to the neoadjuvant treatment (27%). The cCR rate was 10% in 256 patients who received CRTn and 17% in the 130 patients that received CRTn followed by surgery and achieved pCR. The demographic and clinical characteristics and the initial staging of both groups are described in Table 1.

**Table 1:** Demographic and pretreatment clinical characteristics

	Group 1	Group 2	*p* value
	cCR	pCR	
*n* patients (%)	26 (54.2)	22 (45.8)	
Gender (M/F)	13/13	8/14	
Age (years), median (range)	59 (32–82)	55 (26–73)	0.2975
Distance from anal verge (cm), mean (range)	5 (1–12)	7 (2–12)	0.32
T1–2, *n* (%)	8 (31)	4 (18)	0.5035
T3–4, *n* (%)	18 (69)	18 (82)	0.5035
cStage III (N+), *n* (%)	10 (38)	16 (72)	0.04
CRM+, *n* (%)	7 (27)	13 (59)	0.0515
EMVI+, *n* (%)	4 (15)	11 (50)	0.0235
LPLN+, *n* (%)	5 (19)	3 (14)	0.8969
CEA, > 5 ng/dl, *n* (%)	3 (11)	4 (18)	0.8108
Induction XELOX 3 courses, *n* (%)	6 (23)	5 (23)	0.7521
Median follow-up—months (range)	47 (6–94)	49.5 (3–112)	0.7848

*cCR* clinical complete response, *pCR* pathological complete response, *F* female, *M* male, *N+* lymph nodes suspected of malignancy, *CRM+* circumferential resection margin involved, *EMVI +* extramural vascular invasion, *LPLN* lateral pelvic lymph nodes, *CEA* carcinoembryonic antigen, *NS* not significant

Group 1: The decision to opt for non-operative management was motivated in 22 cases (65%) by the impossibility of preserving the annal sphincter and in 4 cases (15%) by the patient’s decision even with the possibility of preservation. Assessment of response between the 6th and 12th week after CRTn was done in 17 patients (65%), the remaining 9 patients (35%) after 12 weeks. The median follow-up time was 47 months, ranging from 6 to 108 months. In three patients (11.53%), an almost complete response was initially observed; for this reason, a full-thickness conventional transanal local excision of the rectum wall over the scar area was performed, finding only fibrosis in the histopathological study and the patients continued in WW. Two patients (8.3%) experienced regrowth of the tumor, both on the luminal surface. One of them presented an ulcerated lesion on the tumor scar after 9 months of follow-up. A biopsy of the lesion was performed due to a high suspicion of malignancy and histologically confirmed. In another case, an irregular indurated lesion that was detected on the scar was completely resected after 10 months of follow-up, which was confirmed as pT2 pNx by histopathology. An abdominoperineal resection was performed as salvage surgery in both patients and are currently disease-free at 56 and 32 months respectively of postoperative follow-up. These were the only two cases that required salvage surgery. One patient (3.8%) experienced distant progression, after 4 months in non-operative strategy and has been on systemic chemotherapy for 31 months, maintaining cCR of the primary tumor since the end of radiotherapy. At the time of this analysis, 24 patients are alive; there were two no-related cancer death.

Group 2: the response assessment between the 6th and 12th week post CRTn (range 4–12 weeks) was done in 14 patients (63.63%). Median interval time since the end of radiotherapy to surgery was 15 weeks (range: 9–34 weeks). The surgeries performed were anterior resection in 19 cases (86%), intersphincteric in two (9%), and abdominoperineal resection in one (5%). Median follow-up time was 49.5 months, with a range of 3 to 112 months. During follow-up, local recurrence was observed in one of the 22 patients (4.8%), 12 months after intersphincteric resection, affecting sectors of the perineum and inguinal areas, not amenable to salvage surgery. The patient died after 15 months of follow-up. Distant recurrence was observed in three patients (19.2%). One of them presented pulmonary and hepatic metastases after 36 months of follow-up and two other patients exhibited lung metastases at 6 and 26 months after surgery. Two of the three patients died of the disease. At the time of the analysis, 19 patients are still alive.

**Fig. 2:**
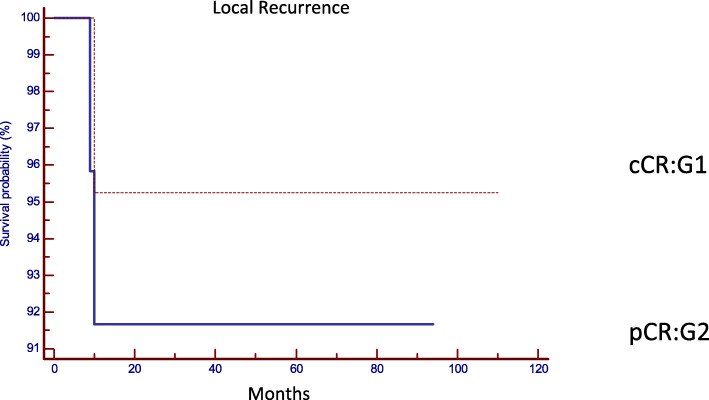
Local recurrence (*p* = 0.6235, HR: 1.8004, 95% IC 0.1867 to 17.3630). cCR clinical complete response and pCR pathological complete response

**Fig. 3:**
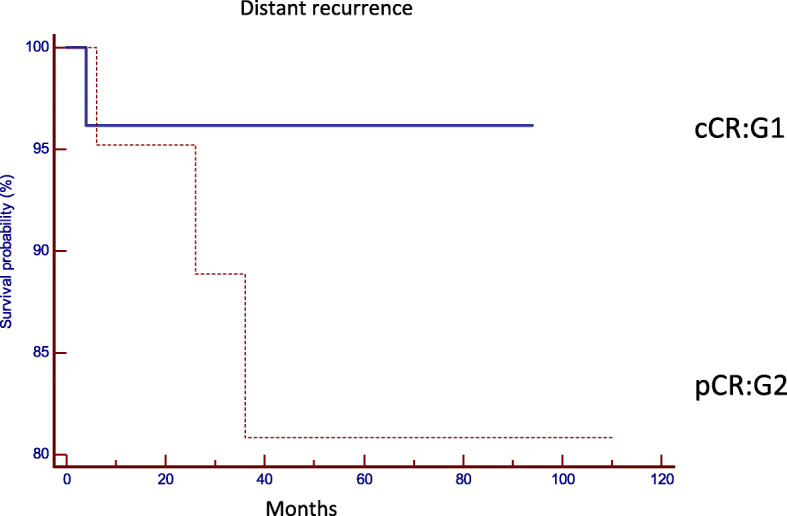
Systemic recurrence (*p* = 0.2237, HR: 0.2700, 95% IC 0.03763 to 1.9381). cCR clinical complete response and pCR pathological complete response

**Fig. 4:**
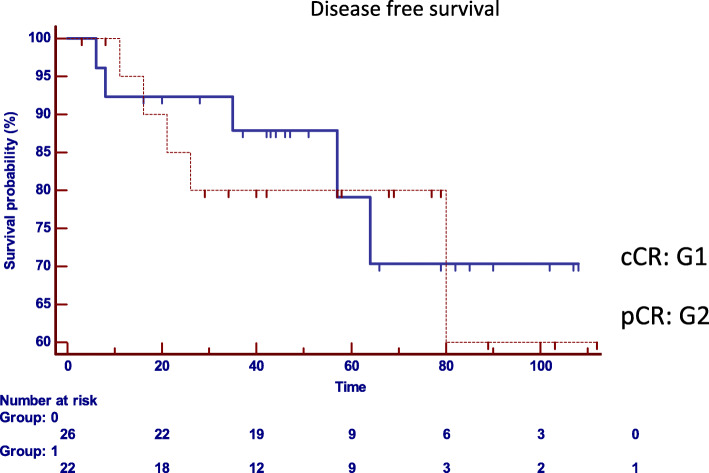
Disease-free survival (*p* = 0.7546, HR: 0.8213, 95% CI 0.2363 to 2.8541). cCR clinical complete response and pCR pathological complete response. Group 0: Group 1 cCR; Group 1: Group 2 pCR

**Fig. 5:**
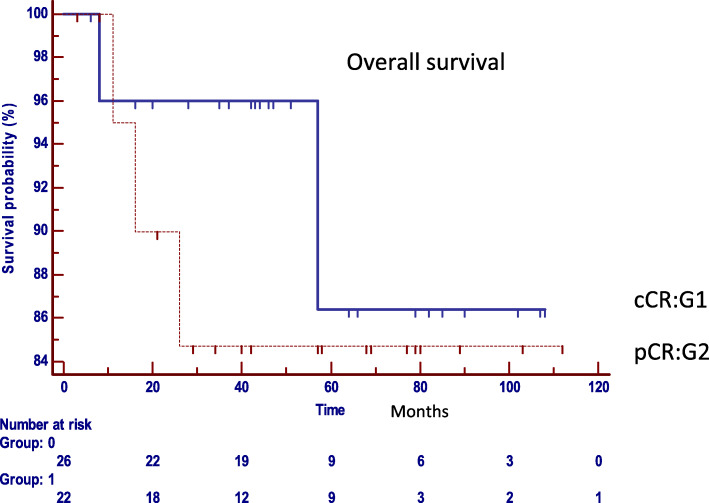
Overall survival (*p* = 0.5367, HR: 0.5737, 95% CI 0.09890 to 3.3280). cCR clinical complete response and pCR pathological complete response. Group 0: Group 1 cCR; Group 1: Group 2 pCR

The comparative analysis between both groups with regard to the oncological results confirmed that there are no statistically significant differences in the local or distant control in both strategies (Table 2). Local recurrence was observed in two patients in G1 (8.3%) and in one patient in G2 (4.8%), (*p* = 0.6235, HR: 1.8004, 95% IC 0.1867 to 17.3630) (Fig. 2), distant recurrence was observed in one patient in G1 (3.8%), and in three cases in G2 (19.2%). (*p* = 0.2237, HR: 0.2700, 95% IC 0.03763 to 1.9381) (Fig. 3). Disease-free survival was 87.9% in G1 and 80% in G2 (*p* = 0.7546, HR: 0.8213, 95% CI 0.2363 to 2.8541) (Fig. 4). Overall survival was 86% in G1, and 85% in G2 (*p* = 0.5367, HR: 0.5737, 95% CI 0.09890 to 3.3280) (Fig. 5). Initial staging data for patients with local or distant progression in both groups are presented in Table 3.

**Table 2:** Oncological outcomes. Statistic analysis

	Group 1	Group 2	*p* value
	cCR	pCR	
*n* patients	26	22	
Local relapse/regrowth (*n*/%)	2/8.3%	1/4.8%	0.6235
Distant recurrence (*n*/%)	1/3.8%	3/19.2%	0.2237
Disease free survival (%)	87.9%	80%	0.7546
Overall survival (%)	86%	85%	0.5367

*cCR* clinical complete response, *pCR* pathological complete response

**Table 3:** Clinical initial staging and surgery in patients who developed systemic or local relapse

Group	Distance from AV	T	*N*	CMR	EMVI	LPLN	Induction	Adjuvant	Relapse	Rescue
							CT	CT	Surgery	
1cCR	3	3	0	Free	No	No	No	No	Local	APR
1cCR	3	3	1	Free	No	No	No	Yes	Local	APR
1cCR	2	4b	0	Involved	No	No	No	No	Systemic	–
2pCR	2	4b	0	Involved	No	No	No	No	Local	No
2pCR	10	3b	1	Free	Present	Present	No	Yes	Systemic	–
2pCR	3	3c	2a	Free	No	No	No	Yes	Systemic	–
2pCR	3	4b	0	Involved	No	No	Recived	no	Systemic	–

*cCR* clinical complete response, *pCR* pathological complete response, *AV* anal verge, *CRM* circumferential resection margin, *EMVI* extramural vascular invasion, *LPLN* lateral pelvic lymph nodes, *CT* chemotherapy, *APR* abdominoperineal resection

## Discussion

The option of organ preservation by implementing a WW strategy in cCR after neoadjuvant therapy has increasingly become an attractive approach after being observed in several studies in high-volume centers that it has similar oncological results as surgery with pCR [3, 5, 16]. Functional results are added to the benefits of this strategy in avoiding morbidity and mortality resulting from surgery, as we have found in a study conducted by our team that is currently in press, in which we observed an improved the quality of life and reduced incontinence in WW patients.

Although WW has been evaluated as being viable and safe, the determination of response continues to be one of the major debate topics, as well as the eligibility criteria of the patients who could opt for this option, and the feasibility of a systematized follow-up that could allow access to the patients to a salvage surgery in the case of a change in their clinical condition [9, 22, 23, 25–31]. Because not always it is possible to detect the clinical complete responders some patients need to be operated and showed a complete pathological response (ypT0N0) like our patients in G2. Additionally in a small number of them, the surgery was performed in spite of suspected and probable clinical complete response because they could undergo to sphincter preservation surgery.

Initially in our multidisciplinary committee called “Co-recto,” we have cooperatively reviewed results in complete responders patients, with other centers in the Autonomous City of Buenos Aires, in which we observed that the oncological results are good and similar in patients with cCR and WW and with pCR. In this work, patients from a single institution have currently been evaluated [23, 32].

One of the factors that we consider of great relevance to determine the response to the treatment is to establish the best time to perform re-assessment; diverse studies have proposed from 3 to 24 weeks after the end of radiation therapy, which reflects the lack of strict consensus in this regard [3, 6, 22, 29], performing digital rectal examination, rigid proctosigmoideoscopy, abdominal and pelvic MRI, and chest CT scan. In our team, we preferred to conduct the evaluation of response at least 6 weeks after the end of neoadjuvant therapy, and in those cases with a very good response, we continued with periodic monthly reevaluations, thus postponing surgery. We decided to wait longer, thinking of the possibility of an intentional WW strategy, especially in the most distal tumors. In two of our patients, who were on an abdominoperineal resection plan, and had to wait for more than 12 weeks, the treatment had only shown a scar lesion. Hence, the indication for surgery was changed to a non-operative management. Moreover, MRI also showed cCR, and the morbidity and mortality of surgery were avoided, providing the additional benefit of sphincter preservation, without definitive stoma.

We did not perform an endoscopic biopsy of the residual lesion as a detection or confirmation tool for complete responders because, in general, the tumor involvement of the mucosa and the submucosa is modified and replaced by scar tissue, and at this level of the rectal wall, only fibrosis and inflammatory processes are often found. The histopathological study of proctectomy specimens post chemoradiotherapy may confirm the absence of atypical cells in the mucosa and submucosa, both of them replaced by fibrosis and persistence of residual tumor in the periphery at a muscular layer level itself or at extramural level, concluding the histopathological staging as ypT2 or ypT3. For these reasons, in our experience, a negative endoscopic biopsy after CRTn does not at all justify to rule out the need for radical resective surgery [28, 29].

Conventional or minimally invasive local transanal resection (TAMIS) is an option when a minimal residual lesion or almost complete response are suspected at re-staging time. This procedure was performed in three of our cases, allowing us to conduct a histopathological study of the full width of the rectal wall. This method has the limitation that any lateral spreading remained foci could be left outside the demarcated limits in the resection; the follow-up must be exhaustive when the result of the scar study was negative. In case of detecting a local recurrence, the salvage surgery can be immediately proposed [3, 6, 7, 22, 33].

Our local recurrences were detected on the luminal surface; we observed tumor regrowth in two of our patients in G1 (8.3%), both within the first year of follow-up, coinciding with the times reported in the literature but the rates of regrowth reported were ranging from 10 to 22.1% [6, 7, 22, 23], higher than that observed in our series. Although we had a low rate of regrowth, we could not perform sphincteric preservation surgery at surgery salvage time, because of tumor location with respect to the sphincter complex. In 2018, the experience of the International Multicenter Registry, WW International Database (IWWD) was published [9], which included 880 patients with cCR and the local recurrence/regrowth was 25.2% (95% CI 22·2–28.5%), most of whom were within the first 2 years and 97% in the intestinal wall. Maybe this difference reflects the strict patient eligibility criteria for the WW strategy of our team, the MRI systematization of response evaluation, and careful follow-up.

With regard to the distant progression of the disease, there were no statistically significant differences between the two groups, although we observed a greater tendency of distant metastases in G2 pCR. This difference is probably unrelated to the strategy implemented but related to the systemic risk characteristics of the initial staging, which are different between G1 and G2, or intrinsic molecular characteristics. The molecular predictor factors of response to CRTn are currently being studied and analyzed by our study group [34, 35].

Our results coincided with those of other groups that showed no differences between both strategies. Habr-Gamma et al. [26] published that in 173 patients treated with CRTn, there was 39% of cCR (67 cases), in which a large number of them were in initial clinical stages (I 16%, II 63%, and III 21%), the local recurrence was observed in 8 patients (11%) and distant recurrence in 7 patients (10%) [4, 25, 26, 36]. Unlike these series, even in locally advanced tumors with higher initial risk (69% T3-4 and 36% N+), we observed that the WW strategy was safe in local and distant control and with good overall survival. Furthermore, despite this difference in initial staging, we observed good local recurrence rates of 8.3% and 3.8% of distant progression for complete clinical responders. We also noted a similar percentage for distant metastases in the international multicenter IWWD analysis, 8,1% (95% CI 6·2–10·5), in 71 of the 880 patients included, 54% were initially staged as T3-4, and 50% had lymph node involvement. We also coincided in the higher frequency of pulmonary rather than hepatic secondarism [9]. These observations contrast with the only series of publications of the Memorial Sloan Kettering Cancer Center (MSK) [27] in which they compared 113 cases in the WW strategy with 136 cases with pCR, showing worse overall survival at 5 years in the WW group (73% vs. 94%), justified by a higher rate of metastatic disease in those patients who experienced regrowth of the tumor.

The overall survival in our study was 86 and 85% respectively for each group, thus confirming that our results are within the international figures mentioned above. One of the first comparative series published by Habr-Gamma et al. showed no differences between their group under observation and those of operated patients, which was 100% and 88%, respectively (*p* = 0.01) [3]. Further analyses, such as this systematic review of 17 articles and 2973 cases, showed an overall survival of 93.5% [6] after 3 years of follow-up in patients under WW strategy. It is known that patients with pCR have better survival rates and a lower recurrence rate compared with resected patients presenting residual tumors [4].

When analyzing the impact of time until post CRTn surgery on the pCR rate, we observed that our waiting times are longer than 12 weeks due to patient-related factors, such as sociocultural and economic barriers in our public care setting, which is one of the greatest difficulties that we face, and despite this fact, we did not have higher percentages of pathological complete responders than in the different published articles on the subject (17% of our operated post CRTn patients). The Grecca-6 trial [25] on the analysis of 265 patients from 24 medical centers, compared the percentage of pCR, by performing surgery on the 7th week or after the 11th week after CRTn and observed a percentage of 15 and 17.4% respectively without significant differences (p:NS) [4, 26]. In contrast, Petrelli et al. [37], in a meta-analysis on 13 analyzed works and a total of 3584 patients, observed a higher percentage of pCR in those who underwent surgery later than 8 weeks post CRTn (19.5%) than in those patients operated before 8 weeks (13.7%).

Our work has limitations, such as the fact that it is a retrospective review, with a limited number of patients, WW strategy was not achieved intentionally but “accidentally,” times assessment that were influenced by the characteristics of clinical practice in public health, and the impacts of its fragmentation, for example, performing radiation therapy, imaging, and chemotherapy in different medical centers. However, one of its strengths is that patients are strictly followed-up, receiving the same personal attention and systematized evaluation.

In conclusion, surgery can safely be deferred in patients with a complete clinical response; our study shows that there are no differences in local and distant recurrence and overall survival between both strategies, with similar long-term results, with the added benefit of organ preservation in the WW group, which we consider safe and with very good impact on the quality of life, always maintaining a systematized and consistent follow-up.

## Data Availability

The datasets used and analyzed during the current study are available from the corresponding author on reasonable request.

## Abbreviations

TME: Total mesorectal excision
CRTn: Neoadjuvant chemoradiotherapy
cCR: Clinical complete response
pCR: Pathological complete response
WW: Watch and wait
MRI: Magnetic resonance imaging
VCC: Videocolonoscopy
EMVI: Extramural vascular invasion
CEA: Carcinoembryonic antigen
CRM: Circumferential resection margin
CT: Computed tomography
